# Health-related quality of life of migrant workers: a systematic literature review

**DOI:** 10.1186/s12889-023-15981-5

**Published:** 2023-05-30

**Authors:** Hyun-Jin Cho, Kyoungrim Kang, Kyo-Yeon Park

**Affiliations:** 1 College of Nursing, Pusan National University, Yangsan-si, South Korea; 2 College of Nursing, Research Institute of Nursing Science, Pusan National University, 49 Busandaehak-ro, Mulgeum-eup, Yangsan-si, Gyeongsangnam-do 50612 South Korea

**Keywords:** Migrant workers, Health-related quality of life, Healthcare, Systematic literature

## Abstract

**Background:**

Currently, the number of migrant workers residing in Korea is continuously increasing, which is exacerbating the workforce shortage in its society. Migrant workers experience health problems or stress due to rapid environmental changes, consequently impairing their quality of life (QoL). Accordingly, this literature review aimed to prepare basic data by identifying factors related to the health-related quality of life (HRQoL) of migrant workers in Korea.

**Method:**

In total, the literature search used seven databases to find all documents corresponding to related subject words until June 7, 2022, including PubMed, Cumulative Index to Nursing and Allied Health Literature, Embase, Regional Information Sharing Systems, Korean Medical database, Science ON, and DataBase Periodical Information Academic. Furthermore, this study used the Cochrane Library and Google Scholar to manually search, to include comprehensive literature. Moreover, both English and Korean were used to search for the main terms.

**Results:**

In total, nine articles were selected. The World Health Organization Quality of Life Brief Version tool was used in six studies to measure HRQoL. Factors affecting the HRQoL of domestic migrant workers included general characteristics such as monthly income and residence period, physical and psychological health-related characteristics such as health promotion behaviour, medical service satisfaction, and depression, and social factors such as social support and cultural adaptation stress. Social support was an important variable affecting the QoL. Particularly, increased social support improved health-related QoL. In addition, higher medical service satisfaction and lower cultural adaptation stress increased HRQoL.

**Conclusions:**

Social factors such as social support and cultural adaptation stress affect the HRQoL of migrant workers. Therefore, the social integration program should be expanded to ensure that migrant workers can adapt to the domestic culture at an early stage. In addition, people require active support to improve the QoL in Korea through activities such as self-help groups to help them cope with stressful situations and experience positive emotions. Moreover, it is necessary to provide information on domestic medical services as well as support for medical information for self-health management to improve the quality of medical services for migrant workers.

## Background

Migrant workers began entering the country in the 1980s because of labour shortages in domestic industrial sites and the increased prevalence of aging and avoidance of risky industries [1]. Consequently, migrants have become established as an indispensable workforce in the domestic labour scene [2]. In October 2019, approximately 2.48 million foreigners were residing in Korea, of which 580,000 of them were seeking employment. Furthermore, Korea has reported 2.25 million illegal aliens and a steady increase in the number of migrant workers per annum [3]. Thus, this can contribute to economic development by solving Korea’s workforce shortage.

However, many migrant workers perceive linguistic and cultural differences and experience maladaptation due to social alienation [4]. In addition, studies have found that the poor working environment and discriminatory conditions, such as unstable employment patterns and long hours of work, negatively affect the mental health of migrant workers, leading to a decrease in the quality of life (QoL) [5–8]. Furthermore, situations that violated the legal rights of migrant workers (e.g., housing conditions and low wages) comprised factors that severely impaired the QoL [9] of migrant workers who experienced various difficulties. Thus, it is necessary to prioritise their health-related quality of life (HRQoL).

QoL is a subjective evaluation based on how individuals feel about life, including positive and negative emotions [10]; life satisfaction is one of the components of QoL [11]. The QoL of migrant workers is determined by their subjectively perceived life satisfaction and the positive or negative emotions they experience while living in Korea for employment [9]. Moreover, QoL includes subjective evaluations in various life areas such as work, housing, and health, while HRQoL is a subjective evaluation centred on health such as physical and emotional health and function [12].

Research has found that the acculturation stress that occurs when migrant workers adjust to Korean society causes mental health problems such as depression and anxiety, and negatively affects the life satisfaction of migrant workers [13]. Furthermore, subjective health status, average monthly income, and working hours were identified as factors affecting the HRQoL of migrant workers in Korea [5, 7, 9]. Migrant workers with higher HRQoL exhibit increased productivity, which can facilitate economic development in Korea. In addition, it is necessary to manage the HRQoL of migrant workers beyond the individual level, at the social and national levels to guarantee their basic rights [7, 9]. HRQoL can affect the priority of resource utilization and decision making regarding mid- to long-term health care among migrant workers. Therefore, it is necessary to comprehensively understand HRQoL.

The present systematic review of literature can facilitate the establishment of a reliable research base to improve the health of migrant workers in Korea and seek ways to improve their HRQoL. Therefore, this study aims to identify the overall research trends by systematically examining previous literature on HRQoL investigating migrant workers in Korea. Consequently, this study intends to provide basic data for HRQoL programs for migrant workers in Korea.

### Purpose

The purpose of this study is to assess the HRQoL of migrant workers in Korea and prepare basic data for developing a program to improve HRQoL by identifying factors related to migrant workers’ HRQoL. Furthermore, this study aims to systematically review data and attain two specific goals.

First, the general characteristics and research trends on the HRQoL of migrant workers in Korea are identified.

Second, factors related to the HRQoL of migrant workers in Korea are identified.

## Methods

### Study design

A systematic literature review was conducted to identify factors related to the HRQoL of domestic migrant workers and to synthesise studies.

The present study was conducted based on the systematic literature review reporting guidelines of the Preferred Reporting Items for Systematic Reviews and Meta-Analysis (PRISMA) group [14] and the systematic literature review manual presented by the National Evidence-based Healthcare Collaborating Agency (NECA) [15].

### Key questions

The Participants, Intervention, Comparison, Outcome (PICO) framework was established for systematic literature review. However, intervention (I) and control group (C) were excluded from the PICO because this study was not conducted to synthesise the effects of a specific intervention. The literature search began by setting the study participants (P) and outcome (O). Specifically, the participants (P) were migrant workers in Korea, while the outcome (O) was their HRQoL.

### Literature search, collection, and selection process

#### Literature search

The current study searched literature published until June 7, 2022. Electronic databases were searched for all studies corresponding to related keywords without restriction on the year of publication. For the literature search, seven databases were searched: PubMed, Cumulative Index to Nursing and Allied Health Literature (CINAHL), Embase, Research Information Sharing Service (RISS), Korean Medical database (KMbase), Science ON, and DataBase Periodical Information Academic (DBpia). To increase the sensitivity of the literature search, grey literature was manually searched using Cochrane Library and Google Scholar. Furthermore, additional literature was searched by reviewing the reference lists of the studies obtained through the database search. Particularly, the main keywords in the databases were searched in English and Korean. English search terms were “health-related quality of life” OR “quality of life” OR “HRQoL” OR “QoL,” “migrant workers” OR “foreign workers” OR “migrant labours” OR “migrants” OR “transients,” OR “Korea.” Furthermore, each keyword was connected by the term “AND,” and the search was conducted based on the characteristics of each database. Moreover, Korean search terms were searched using “quality of life” OR “health-related quality of life,” “migrant worker” OR “foreign worker”.

#### Literature collection and selection

**Fig. 1:**
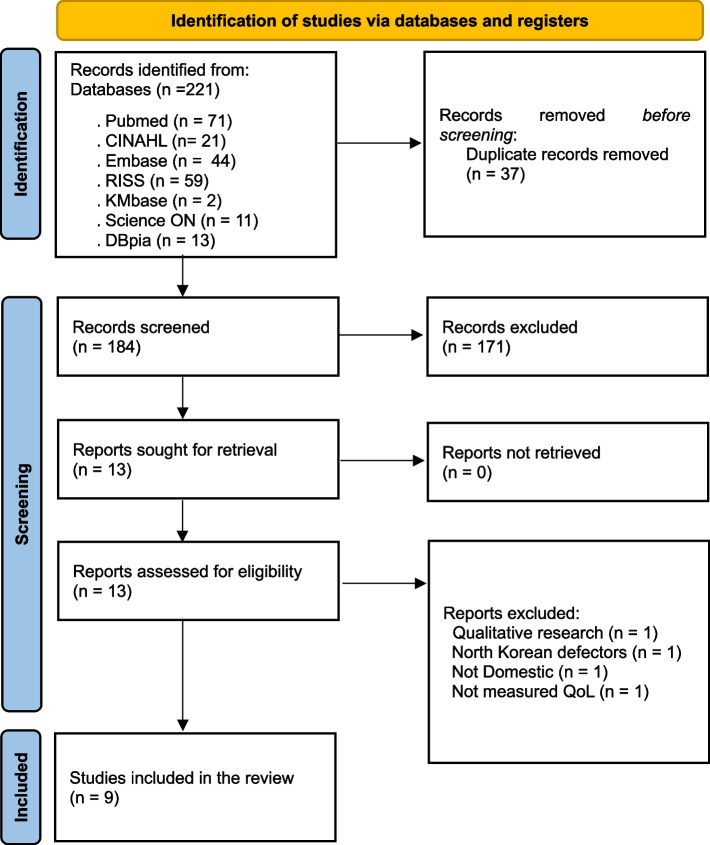
The study selection process using PRISMA 2020 Abbreviations: PRISMA = Preferred Reporting Items for Systematic reviews and Meta-Analysis; CINAHL = Cumulative Index to Nursing and Allied Health Literature; RISS = Research Information Sharing Service; Kmbase = Korean Medical database; Dbpia = DataBase Periodical Information Academic; QoL = Quality of Life

Studies were collected using electronic databases, and the collected literature was managed using EndNote X9.3.1 (compatible with EndNote 20), a bibliographic management program. The literature selection for the review was performed according to the reporting guidelines recommended by PRISMA 2020 Statement. The inclusion criteria were (1) studies on migrant workers in Korea, (2) studies in Korean or English, and (3) studies published in academic journals. Whereas, the exclusion criteria were (1) studies on migrants other than migrant workers, (2) qualitative studies, and (3) conference presentations, abstracts only, dissertations and reports. The literature search yielded 221 studies, including 71 from PubMed, 21 from CINAHL, 44 from Embase, 59 from RISS, two from Kmbase, 11 from Science ON, and 13 from DBpia. Using EndNote, 37 duplicate papers were identified and removed. For the remaining 184 articles, titles and abstracts were reviewed according to the inclusion and exclusion criteria, while 171 studies that did not fit the study purpose were excluded. Among the 13 selected studies, there were no conference presentations, papers or reports, and if there were only abstracts, full texts could be requested through the library to check all the studies. After an in-depth review of the full texts of the selected 13 studies, this study excluded one qualitative study, one study on North Korean defectors, one study on foreign migrant workers, and one study that did not use QoL tools. Consequently, nine studies were selected for the systematic review and have been identified in full text. Three researchers independently performed the literature selection process to ensure the validity and reliability of the results. Each researcher went through an independent review and prepared a result table in a unified format. Subsequently, the researchers examined the results in a team meeting every two weeks and cross-reviewed the selected literature. During this process, researchers addressed disagreements by reviewing the manuscript through a research meeting and adjusting until an agreement was reached. The literature excluded in the selection stages was recorded and the document selection process was described using the 2020 PRISMA systematic review flow chart [16] (Fig. 1).

### Quality assessment

The quality evaluation of the literature was performed using the Effective Public Health Practice Project tool for quantitative research [17]. This tool comprises six domains to be rated “strong (1),” “medium (2)”, and “weak (3),” namely, selection bias, study design, confounders, blinding, data collection methods, study withdrawal, and drop out. Thereafter, the overall rating and the final decision of the reviewers were evaluated as “strong (1),” “medium (2),” and “weak (3).” Three researchers independently evaluated six areas of the final literature selected in this study. One of the three researchers who participated in the study conducted a number of systematic literature review studies and quality of life studies, taught courses related to systematic literature review and meta-analysis for many years, and are currently conducting a systematic literature review studies. In addition, two researchers also had experience participating in systematic literature review studies, systematically learned lectures on systematic literature review and meta-analysis in the doctoral course, and conducted several studies related to quality of life. Finally, the overall grade and final evaluation were made after discussions.

### Data extraction

By analyzing the characteristics of the literature included in the review among the variables related to the author, publication year, study design, study subjects (i.e., number, gender, age, and country), QoL measurement tool, and HRQoL, factors identified as statistically significant were extracted and organised in a table.

## Results

### Characteristics of the included literature

A total of nine studies were included in this review. Table 1 shows the general characteristics of the literature. Two studies were published in 2018 and 2019 (22.2%), while one study was published in 2008, 2012, 2013, 2014, and 2020 (11.1%). The study design consisted of two descriptive survey studies (22.2%), six correlation studies (66.6%), and one intervention study (11.1%) that provided clinical art therapy. All nine studies (100.0%) focused on migrant workers. The average age of the participants in one study (11.1%) was between 20 and 30 years, while three studies (33.3%) included participants aged between 30 and 40, and two studies (22.2%) comprised participants between 40 and 50 years. One article (11.1%) did not have information on age while two articles (22.2%) did not specify the exact average age. The samples comprised two studies (22.2%) with a maximum of 100 people; four studies (44.4%) with a minimum of 100 and a maximum of 200 people; one study (11.1%) consisted of at least 200 to 300 people; one study (11.1%) with at least 300 to 400 people, and one study (11.1%) with at least 400 to 500 people. Seven studies (77.7%) specified the nationalities of the study participants, including China, Indonesia, Mongolia, Philippines, Nepal, Vietnam, Sri Lanka, Cambodia, Pakistan, and Bangladesh. Three studies (33.3%) investigated single-national migrant workers of China, Indonesia, and Mongolia. Among them, most of the migrant workers were Chinese, which was consistent in four studies. Furthermore, five studies (55.6%) specified the occupations of migrant workers and found that most of them were factory workers. The studies also found factory, construction, agricultural and livestock, and service workers. Five studies indicated that the participants resided in Seoul or the suburbs of Seoul, two studies comprised participants in Busan or the suburbs of Busan, as well as Seoul and Busan in one study. Furthermore, 1 study consisted of participants from 10 cities including Seoul and Busan. Moreover, two studies specified whether visa registration was required, while seven studies did not specify visa registration status. In addition, six studies specified the migration period whereas three studies did not specify the migration period. The World Health Organization Quality of Life Brief (WHOQoL-BREF) Version 6, SF-36-K, SF-12v2, and Concise Measure of Subjective Well-Being were each found in one study. Four studies published the questionnaire in Korean, three studies were published in native languages, one study was in Korean and English, and one study’s language was unspecified.

**Table 1:** Characteristics of literature investigating migrants in Korea

Author (year)	Nationality (N)	Study design	Sample N (male %) Mean age (SD) Region of residence	Migration period (Year) Visa registration status (N, %)	HRQoL Assessment / Language	Correlates included in the study / Variables examined (Instruction)	Key findings	EPHPP score
Kim et al. (2008) [18]	China (93), Indonesia (15), Vietnam (8), Mongo (7), Philippines (5), and others (5)	Descriptive	133 (32.3%) 46.4 (13.66) Seoul, Gyeonggi	Not reported Industrial training 22 (16.6%) Illegal 111 (83.4%)	SF-36-K /Korean	ANONA / Demographic factors	There was a significant difference in HRQoL according to the age of participants (*F* = 3.852, *p* = .013), and it was the highest for those under the age of 35. There was no significant difference according to gender, education, disease, smoking & drinking status, and income. Physical health and mental health were significantly correlated (*r* = .663, *p* < .001).	3
Shin (2012) [19]	Mongo	Descriptive	453 (60.9%) 26.5 Seoul, Busan, Daegu, Daejeon, Ulsan, etc.	Not reported Legal 333 (73.5%) Illegal 120 (26.5%)	WHOQoL-BREF /Korean	Path analysis / Job satisfaction, social support, hope	The QoL of illegal immigrant Mongolian migrant workers was significantly lower than that of legally resident Mongolian migrant workers (*p* = .005). The higher the job satisfaction (*p* < .001), the higher the social support (*p* < .001), the higher the hope (*p* < .05), the better the QoL. Hope played a significant mediating role in the path from job satisfaction and social support to QoL(*p*).	3
Park et al. (2013) [20]	Not reported	Experimental	Exp.8 /Cont.8 (Not reported) Not reported Seoul	Not reported Not reported	WHOQoL-BREF /Korean	ANCOVA / Depression (BDI), anxiety (BAI), DAP	Clinical art therapy reduced the depression and anxiety of migrant workers, improved QoL, and there was a statistically significant difference (*p* < .05).	2
Lee et al. (2014) [7]	Not reported	Cross-sectional, Descriptive correlation	135 (77.0%) 20–30(years) = 68.1% 31–40(years) = 14.8% ≥ 41(years) = 17.1% P region, Urban, Rural	< 1 = 50 1≤~<2 = 26 2≤~<3 = 23 ≥ 3 = 36 Not reported	WHOQoL-BREF /Korean	Regression (hierarchical) / Status of medical service use, satisfaction with medical service	The longer the period of residence in Korea (*F* = 2.39, *p* = .041) and the higher the satisfaction with medical services (*F* = 4.28, *p* = .001), the higher the QoL.	3
Kim et al. (2018) [9]	China (112), Nepal (94), Sri Lanka (40), Cambodia (34), Pakistan (21), Philippines (17), and others (35)	Descriptive correlation	355 (62.8%) 36 Gyeonggi	< 1 = 25 1≤~<3 = 105 3≤~<5 = 110 ≥ 5 = 115 Not reported	Concise Measure of Subjective Well-Being /National	Regression (multiple) / AS	Age was 50–59 (*F* = 2.83, *p* = .025), education level was middle school graduation (*F* = 3.05, *p* = .048), better health (*F* = 3.67, *p* = .006), the period of residence was more than 5 years (*F* = 4.09, *p* = .003), the higher housing satisfaction (*F* = 7.33, *p* < .001), the higher monthly income (*F* = 3.85, *p* = .010), the higher the frequency of contact with Korean friends (*F* = 3.70, *p* = .047), the higher the QoL. The higher the AS, the lower the QoL of migrant workers (*p* < .05).	3
Lim (2018) [21]	China, Nepal, Mongo, and 6 countries	Descriptive correlation	179 (90.8%) 35 Seoul, Gyeonggi	Mean 4.3 Not reported	WHOQoL-BREF / Not reported	Regression (multiple) / Acculturation type (EAAM)	Integration (*r* = .47, *p* < .001) and assimilation (*r* = .43, *p* < .05) scores increased, and QoL increased, but marginalization was negatively correlated with QoL (*r* =-.37, *p* < .001). As a result of regression analysis, age, monthly income, years in Korea, integration, and separation were important predictors for the QoL of migrant workers, and they had an explanatory power of 74.0%.	3
Dineva et al. (2019) [22]	Indonesia	Cross-sectional, Correlational	91 (76.9%) 20–29(years) = 82.4% ≥ 30(years) = 17.6% Seoul, outside Seoul	< 1 = 25 1≤~<2 = 37 3≤~<5 = 16 ≥ 5 = 13 Not reported	WHOQoL-BREF /National	Regression (multiple) / AS, depression (PHQ-9), individual factors, environmental factors (social support and organizational support) (MSPSS, POS)	Acculturative stress (*r* =-.42, *p* < .001) and depression (*r* =-.47, *p* < .001) had a significant negative correlation between QoL. As a result of regression analysis, social support and acculturative stress were important variables for QoL, and they had an explanatory power of 74.0%.	3
Cho et al. (2019) [8]	Chinese (103), Korean Chinese (125)	Cross-sectional	228 (43.8%) 41.74 (11.49) Seoul, Busan	< 3 = 79 3≤~<5 = 36 5≤~<10 = 77 ≥ 10 = 36 Not reported	SF-12-v2 /Korean, Chinese	Structural equation / HPB (HPLP-II), AS (ASS), OS (KOSS-26), CSN (ICSN), HL (AAHLS), SE (NGSES)	HRQoL had a statistically significant negative correlation with the barriers of AS (*r *=-.249) and OS (*r *=-.268) but showed a positive correlation with ICSN (*r* = .310), HL (*r* = .288), SE (*r* = .449), and HPB (*r* = .503) (respectively, *p* < .001). HRQoL was directly affected by HPB (β = 0.401, *p* = .005), SE (β = 0.186, *p* = .012), and AS (β=−0.176, *p* = .050). OS (β=−0.092, *p* = .008), ICNS (β = 0.091, *p* = .004), and HL (β = 0.093, *p* = .026) had a significant indirect influence on HRQoL, and HPB was a mediating factor. These variables had an explanatory power of 59.1%.	3
Jung et al. (2020) [23]	Philippines (91), Nepal (18), Indonesia (11), Bangladesh (9), and others (23)	Descriptive correlation	152 (76.3%) 34.34 (8.08) Yangsan, Gimhae, Busan	< 2 = 33 ≥ 2 = 119 Not reported	WHOQoL-BREF /Korean, English	Regression (parametric) / Depression (CES-D), social support, HPB (HPLP)	HRQoL was measured by age (*F* = 3.41, *p* = .036), educational background (*t* =-4.05, *p* < .001), monthly income (*F* = 6.37, *p* < .001), period of residence (*t *=-2.77, *p* = .006), Korean language proficiency (*t* = 2.45, *p* = .007), presence or absence of medical benefits (*t* = 2.75, *p* = .007), and perceived health status (*t* =-6.09, *p* < .001). It was found that there was a significant difference. HRQoL was negatively correlated with depression (*r *=-.44, *p* < .001), social support (*r* = .44, *p* < .001) and HPB (*r* = .51, *p* < .001), and there was a positive correlation. The mediating effect of HPB was confirmed to be significant in the relationship between depression (Z = 3.26, *p* < .001) and social support (Z = 3.98, *p* < .001) and HRQoL.	3

*Abbreviations:* *AAHLS *All Aspects of Health Literacy Scale, *AS *acculturative stress, *ASS *Acculturation Stress Scale, *BAI *Beck Anxiety Inventory, *BDI *Beck Depression Inventory, *CES-D *The centre for epidemiological studies-depression scale, *Cont. *control group, *CSN *community storytelling network, *DAP *Draw-A-Person test, *EAAM *East Asian Acculturation Measure, *Exp. *experimental group, *HL *Health literacy, *HPB *Health-promotion behaviours, *HPLP *Health Promotion Lifestyle Profile, *HRQoL *Health-related quality of life, *ICSN *Integrated Connectedness to Neighbourhood Storytelling Network, *KOSS-26 *Korean Occupational Stress Scale-26, *MSPSS *Multidimensional Scale of Perceived Social Support, *NGSES *New General Self-Efficacy Scale, *OS *occupational stress, *PHQ-9 *Patient Health Questionnaire-9, *POS *Perceived Organizational Support scale, *QoL *Quality of Life, *SE *Self-efficacy, *SF-12-v2 *Short Form-12 version 2 heath survey, *SF-36-K *Short Form-36-Korean version health questionnaire, *WHOQoL-BREF *World Health Organization Quality of Life-Bref

### Factors related to the quality of life of migrant workers

Table 1 presents the independent variables indicating the factors that affect the QoL of migrant workers and the variables showing statistically significant differences. The present study analyzed the participants’ general characteristics such as age, monthly income, period of residence, and region of residence, as well as variables of physical and psychological health-related characteristics including hope, depression, and health-promotion behaviours. In addition, social factors such as social support, acculturation stress, job stress, and job satisfaction were studied. As a general characteristic, age was identified as a factor affecting the QoL of migrant workers [21]. Furthermore, Cho et al. [8] and Kim et al. [18] found that age is related to QoL. In terms of monthly income, Kim et al. [9] and Lim [21] showed that higher monthly income indicates higher QoL of migrant workers. The length of residence was confirmed as an influencing factor in the QoL of migrant workers in four studies [7–9, 21]. Particularly, longer periods of residence in Korea indicate higher QoL. Lee et al. found that area of residence showed that the higher the standard of living in the city, the higher the quality of life [7]. Moreover, Kim et al. found that higher housing satisfaction and improved health yielded higher QoL [9]. In addition, Shin [19] found that the legal and illegal status of residence was related to migrant workers’ QoL.

As a health-related characteristic, higher satisfaction with medical services [7] reflected higher QoL among migrant workers. Hope [19] and depression [23] were also confirmed as factors affecting the QoL of migrant workers. Particularly, having hope was confirmed as a mediating effect between job satisfaction and social support that enhances QoL. In addition, Cho et al. [8] and Jung et al. [23] showed that health-promoting behaviours had a significant effect on the QoL. Upon confirming the mediating effect of health promotion behaviours, Jung et al. [23], found that health promotion behaviours partially mediate the relationships between depression and HRQoL as well as social support and HRQoL. Furthermore, a study found that health knowledge or literacy [8] had an indirect effect on HRQoL. Similarly, Park et al. also found that clinical art therapy was related to QoL [20]. As a social factor, social support [19, 22, 23] was identified as an influencing factor on the QoL of migrant workers. Job satisfaction [19], adaptation stress [8, 9, 22], self-efficacy [8], separation, and integration among acculturation styles [21] were also identified as factors affecting the QoL of migrant workers. Moreover, research has found that occupational stress and integrated connection with community storytelling networks [8] had an indirect effect on HRQoL.

## Discussion

The present study was conducted to prepare basic data for improving the HRQoL of migrant workers. Specifically, this study systematically examined the literature identifying factors related to the HRQoL of migrant workers in Korea. Studies investigating the QoL of migrant workers have been conducted steadily since 2008. Among the nine studies included in the final analysis of this review, there were eight descriptive and correlation studies, as well as one clinical art therapy intervention study. However, the present study had limited generalization with small sample size and convenience sampling. Thus, future studies ought to plan and attempt scientific and systematic intervention studies to present a clear explanatory power of factors affecting the QoL of migrant workers and to provide higher-quality evidence-based data. Furthermore, health promotion programs such as group art therapy, holistic health nursing intervention, horticultural therapy, and stretching programs were utilized in a systematic literature review confirming the effectiveness of programs for the health promotion of migrant workers [24]. Moreover, these health promotion programs can be considered in future studies to verify whether they are effective in improving the HRQoL of migrant workers.

The migrant workers were mainly aged between 20 and 50 years. Similarly, the International Labour Organization defined an age distribution of 25 to 54 years old [25] since the study participants were migrant workers, not migrants. Therefore, migrants comprise “the core labour force, which is the age group with the most active labour supply and the highest productivity [25]” and have been employed as migrant workers. The literature indicated that the nationalities of this study’s participants varied, with Chinese (including Korean-Chinese) nationalities occurring the most. Similarly, a study on the health of foreign migrants [26] and foreign workers [27] found that most of the participants had Chinese (including Korean-Chinese) nationalities. Furthermore, since April 2021 [28], most foreign workers were of Chinese nationality (including Korean-Chinese). Thus, a large number of migrant workers with Chinese nationality were included in the study of migrant workers. Moreover, checking the nationality distribution of migrant workers can help compose the program in consideration of culture and language when planning an HRQoL improvement program for migrant workers in the future. In terms of occupations, the migrant workers mostly comprised factory workers and construction workers as research on the QoL of migrant workers were centered in cities such as Seoul and Busan. Thus, agricultural, fishery, and livestock workers are relatively limited. In contrast to cities, rural and fishing villages lacked various benefits in terms of medical, social, and cultural aspects [29]. Furthermore, there were many cases in agriculture, fishery, and livestock industries for migrant workers to apply for workplace health and industrial accident insurance [27]. Therefore, future studies should expand studies on the QoL of migrant workers living in rural areas.

To measure the quality of life of migrant workers, the WHOQoL-BREF [13, 30] tool, a brief version of the WHOQoL-100, was used most frequently. This tool has been translated into multiple languages ​​and has been verified for reliability and validity. Furthermore, the tool consists of physical, psychological, and environmental health, social relations, overall QoL, and general health perception. The WHOQoL-BREF evaluated the QoL and HRQoL in the literature included in this study. Particularly, this tool shows that QoL and HRQoL are not clearly distinguished and utilized. According to the WHO [31], QoL comprises a broad concept affected by an individual’s physical health, psychological state, level of independence, social relationships, and environment. Buchcik et al. [32] stated that physical, psychological, and social dimensions should be included in the definition of QoL and HRQoL. However, no clear distinction was found between the two terms, and the term was selected based on the researcher’s preference. Moreover, the tools of SF-36-K and SF-12v2 were used to evaluate the HRQoL, except for the WHOQoL-BREF in the literature included in this study. The shortened happiness scale (concise Measure of Subjective Well-Being) was also used to evaluate the QoL. The HRQoL of migrant workers comprised a measure that could confirm their level of health and overall well-being for life in Korea [27].

In the case of migrant workers, monthly income and socioeconomic status affected the HRQoL. Therefore, it is necessary to select terms and measurement tools suitable for study in future studies in consideration of these factors. The language of the questionnaires was mainly Korean, while only three studies (33.3%) included questionnaires translated into participants’ native language. In terms of the native language translation questionnaire, two studies consisting of participants with a single nationality and one study (11.1%) underwent systematic translation processes such as reverse translation and cognitive interviews during the translation into their native language. These results suggest that migrant workers have diverse nationalities and that the process of translating the questionnaire into each language is time-consuming and costly. However, when another language is used as the mother tongue, a systematic translation process is required to confirm whether the measurement concept intended by the original tool is appropriate for participants from different cultural and linguistical backgrounds [33]. Therefore, it is necessary to focus on translating the native language tool according to the guidelines to secure the validity and reliability of the research tool in future studies.

The current study found that the general characteristics related to the HRQoL of migrant workers were affected by age, monthly income, period of residence, area of ​​residence, and housing satisfaction. Higher monthly income indicated higher QoL of migrant workers, which was consistent with higher QoL during conditions of high economic levels [34, 35] in a study of factors affecting the QoL. Particularly, this was common in the case of migrant workers who initially wanted to earn money in Korea [36]. Thus, low monthly income could have been associated with a lower QoL. Longer periods of residence in Korea reflected higher QoL because increased migration periods were associated with decreased stress of acculturation due to overcoming language difficulties and acculturation. The acculturation stress was affected by the level of Korean language and the length of stay, which affects mental health and lowers QoL [26, 27]. Thus, the longer the period of residence in Korea, the lower the acculturation stress, which is considered an influencing factor on the HRQoL. In the residential area, the QoL of migrant workers in urban areas was higher than that of migrant workers in rural areas [7]. Particularly, this was because migrant workers in agriculture were exposed to health risks due to a lack of legal protection, skin diseases caused by excessive sunlight exposure, respiratory diseases caused by chemicals and pesticides, and musculoskeletal disorders caused by improper posture and repetitive movements [29].

Migrant workers in the agricultural and livestock industry had relatively low salaries and poor living conditionsin contrast to workers in the manufacturing industry. Thus, it was necessary to improve the living environment of migrant workers as underscored by Kim et al. [9]. The results of this study supported the low HRQoL of migrant workers in rural areas where the agricultural and livestock industries were predominant. Institutional devices may be needed to guarantee the minimum housing rights of migrant workers according to the general characteristics that affect the above-mentioned HRQoL.

Despite the short period of stay in Korea, improving the QoL of migrant workers through the guidance of self-help groups centered on native and intervention programs is necessary to improve their Korean language efficiency to ensure effective adaptation to life in Korea. Furthermore, the QoL of illegal immigrant workers was significantly lower than that of legal immigrant workers as illegal immigrant workers experienced disadvantages in accessing medical and living services due to fear and anxiety regarding deportation [19]. Consequently, the HRQoL of illegal migrant workers is relatively lower than that of their legal counterparts. An increasing trend in the number of migrant workers was observed from 2012 to 2019. Moreover, the employment of migrant workers has become essential in the labour force which has a gradually increasing ratio [37]. Institutional arrangements are needed to ensure that migrant workers have a stable place in Korean society. Particularly, this can be achieved by improving the immigration policy centered on long-term residence-settlement immigration from the labour migration policy that still prohibits settlement to date [28].

Psychological factors such as hope and depression were found to affect health-related characteristics related to the HRQoL of migrant workers. Hope seems to improve the QoL of migrant workers because it provides meaning to life even during difficult circumstances [19] and enhances adaptability by strengthening psychological defense mechanisms [38]. In contrast, depression is a factor that negatively affects HRQoL. Culture shock, homesickness, communication difficulties, and interpersonal stress can threaten the mental health of migrant workers and aggravate depression [23, 39]. A previous systematic literature review [24] showed that psychosocial health promotion programs reduce depression and anxiety among migrant workers. In addition, health promotion behaviour and health knowledge (literacy) were identified as influencing factors that increase HRQoL. In particular, a study found that health promotion behaviour had the most significant impact on HRQoL and it partially mediated the relationship between depression and HRQoL [8]. Therefore, medical information support for the self-management of migrant workers is required. In addition, clinical art therapy is considered effective in reducing depression and anxiety and improving the QoL of migrant workers [20]. Thus, developing and applying health promotion programs is crucial. Furthermore, it is necessary to develop and apply a psychosocial health promotion program that can reduce depression and strengthen hope through such intervention studies. Migrant workers lack medical information, experience communication difficulties when using medical services, and bare the burden of treatment costs which lowers their satisfaction with medical services [7]. Therefore, providing policy support such as developing a platform for providing information on domestic medical services, developing an interpretation program for medical terminology, developing a brochure for multicultural hospitals, and promoting free clinics is crucial.

As a social factor related to the HRQoL of migrant workers, three studies found that social support forms an important variable influencing the QoL [19, 22, 23]. In three studies [19, 22, 23], it was found that social support of migrant workers is a factor that increases health behaviour, and support systems such as medical services increase the quality of life [23]. In addition, by sharing health-related interests and securing a support group that enables health behaviours, migrant workers were able to lower their stress and improve their quality of life through a sense of fellowship and belonging [22]. Similarly, these findings reflect previous studies [40–44] on the relationship between social support and QoL for various groups of people. Particularly, these studies identified social support as a major variable influencing HRQoL. Social support not only improves happiness and reduces stress, but also improves the QoL of migrant workers [45], which consequently improves HRQoL. Therefore, active support is needed to facilitate migrant workers to organise self-help groups to enhance coping with stressful situations and experience positive emotions. Furthermore, three studies found that cultural adaptation stress occurs while adapting to a new culture and has been identified as a factor affecting the QoL of migrant workers [8, 9, 22]. Particularly, the lower the cultural adaptation stress, the higher the QoL. A previous study [9] showed that among the sub-variables of cultural adaptation stress, higher perceived hatred induced higher fear as well as higher levels of social isolation, inferiority, distrust, and communication problems in other domains. Consequently, this reduced the QoL of migrant workers. In contrast, perceived discrimination, homesickness, cultural shock, and guilt were not statistically significant. In terms of the cultural adaptation type, the higher the score of the integration type seeking participation in a new culture while maintaining the identity of one’s own culture, the higher the QoL [21]. Moreover, a study found that the QoL was high in proportion to the integrated connection with the community storytelling network [8]. In the case of migrant workers, non-payment of wages at the workplace, physical and verbal assault, and poor work environment [36] comprised factors that increased the stress of acculturation and were considered to affect their HRQoL. Therefore, it is necessary to expand the types and activities of migrant workers’ support groups to reduce the stress of acculturation in the early stage and to provide information on available support systems in the local community to ensure they can be connected. To solve the difficulties of communication, it is necessary to expand the Korean language education program and use foreigners who are proficient in the Korean language and culture to help them participate in Korean culture while maintaining their own cultural identity. In addition, the social integration program should be expanded to ensure adaptation to the domestic culture by establishing a counseling and support system that can reduce the stress associated with migrant workers’ acculturation.

Upon evaluating the quality of the literature in this study, “about (3)” overall was found because most of the literature included in this review comprised a research study design. Furthermore, the literature was evaluated as low quality. Thus, future experimental studies are required to confirm the effect on HRQoL. In the data collection method, variable measurement tools were mainly used with validated validity and reliability. The selection bias in sampling comprised a nonprobability sampling method where programs or services for migrant workers were provided (i.e., foreign welfare centers, migrant worker centers, Korean language schools, shelters, foreign worker counseling centers, foreign worker support centers, migrant worker free health check-up centers, free clinics for foreigners, etc.). While foreign hospitals, industries, and religious organizations were used for convenience sampling [8, 18, 19, 22] and deliberate sampling [9]. In addition, snowball sampling [22] was selected, where other migrant workers were introduced and investigated through migrant workers participating in the survey. The sampling method was used because it is difficult to determine the location of migrant workers [36] and access is difficult for migrant workers who are reluctant to expose their identity as illegal immigrants [18]. Furthermore, people with difficulties or problems mainly use support groups. However, only 5% of migrant workers use support groups [36]. Thus, the results of the survey investigating migrant workers are more likely to be biased than representative of the population. Moreover, non-stochastic sampling is limited to the representativeness of the sample as mentioned by the literature included in this study [7–9, 18, 19, 23]. Therefore, to secure the representativeness of the population, a probabilistic sampling method should be applied by using the employment permit migrant worker placement table of the Human Resources Development Service of Korea or by using previous studies on the distribution of migrant workers [36] to help to obtain a representative sample.

Finally, this study conducted a systematic literature review to comprehensively identify factors affecting the HRQoL of migrant workers in Korea. Among the final literature selected, only one intervention study was conducted. Furthermore, meta-analysis was not performed because the study characteristics of the nine selected literature were heterogeneous. Therefore, limitations were found in interpreting the study results. However, the results of this study are significant as they provided basic data for developing an HRQoL improvement program for migrant workers by identifying viable intervention resources to improve the quality of life of migrant workers, such as guaranteeing minimum housing rights, long-term residence-settlement immigration policies, mother-in-law self-help groups, Korean language education programs, psychosocial health promotion programs such as clinical art therapy, and a platform for providing information on domestic medical services.

## Conclusion

This systematic literature review attempted to provide basic data to improve the QoL of migrant workers in Korea by identifying factors related to HRQoL. Upon extracting factors related to the HRQoL of migrant workers in the nine studies included in this study, general characteristics including age, monthly income, period of residence, residential area, and housing satisfaction were found. Furthermore, the health-related characteristics included hope, depression, health promotion behaviour, health knowledge (literacy), and satisfaction with medical services, along with social factors including social support, job satisfaction, cultural adaptation stress, and self-efficacy. The literature review indicates that social factors such as social support and acculturation stress affect the HRQoL of migrant workers. Thus, the social integration program such as Korean cultural adaptation programs using their own citizens and psychological counseling to reduce cultural adaptation stress should be expanded to ensure that migrant workers can adapt to the domestic culture at an early stage. In addition, active support is needed to improve the QoL in Korea through activities such as self-help groups to help cope with stressful situations and experience positive emotions. Moreover, it is necessary to provide information on domestic medical services and support for medical information for self-health management to improve the quality of medical services for migrant workers. Based on these results, this review recommends that a study should be conducted to develop an HRQoL improvement program for migrant workers and to verify their effectiveness.

## Data Availability

The datasets generated during and/or analysed during the current study are available from the corresponding author upon reasonable request.

## Abbreviations

QoL: Quality of Life
HRQoL: Health-Related Quality of Life
PRISMA: Preferred Reporting Items for Systematic Reviews and Meta-Analysis
NECA: National Evidence-based Healthcare Collaborating Agency
PICO: Participants, Intervention, Comparison, Outcome
CINAHL: Cumulative Index to Nursing and Allied Health Literature
RISS: Research Information Sharing Service
KMbase: Korean Medical database
DBpia: DataBase Periodical Information Academic
WHOQoL-BREF: World Health Organization Quality of Life Brief Version
